# LivSCP: Improving Liver Fibrosis Classification Through Supervised Contrastive Pretraining

**DOI:** 10.3390/diagnostics15243226

**Published:** 2025-12-17

**Authors:** Yogita Dubey, Aditya Bhongade, Punit Fulzele

**Affiliations:** 1 Department of Electronics & Telecommunication Engineering, Yeshwantrao Chavan College of Engineering, Nagpur 441110, India; adityabhongade24@gmail.com; 2 Directorate of Research & Innovation, Sharad Pawar Dental College, Datta Meghe Institute of Higher Education & Research, Wardha 442001, India; punitr007@gmail.com

**Keywords:** contrastive learning, liver fibrosis, pretraining, supervised contrastive learning, vision transformer

## Abstract

**Background**: Deep learning models have been used in the past for non-invasive liver fibrosis classification based on liver ultrasound scans. After numerous improvements in the network architectures, optimizers, and development of hybrid methods, the performance of these models has barely improved. This creates a need for a sophisticated method that helps improve this slow-improving performance. **Methods**: We propose *LivSCP*, a method to train liver fibrosis classification models for better accuracy than the traditional supervised learning (SL). Our method needs no changes in the network architecture, optimizer, etc. **Results**: The proposed method achieves state-of-the-art performance, with an accuracy, precision, recall, and F1-score of 98.10% each, and an AUROC of 0.9972. A major advantage of LivSCP is that it does not require any modification to the network architecture. Our method is particularly well-suited for scenarios with limited labeled data and computational resources. **Conclusions**: In this work, we successfully propose a training method for liver fibrosis classification models in low-data and computation settings. By comparing the proposed method with our baseline (Vision Transformer with SL) and multiple models, we demonstrate the state-of-the-art performance of our method.

## 1. Introduction

Liver diseases, particularly non-alcoholic fatty liver disease (NAFLD) and fibrosis, are a global health concern. Liver fibrosis is characterized by the excessive accumulation of extracellular matrix proteins, causing chronic liver diseases.

Globally, liver disease contributes for approximately 2 million deaths per year worldwide, with 1 million due to liver cirrhosis and 1 million due to viral hepatitis [1]. The global prevalence of NAFLD among adults is estimated to be around 32%, and higher (40%) in males compared to females (26%) [2]. The global prevalence of alcohol-related liver diseases (ARLDs) was 4.8%, as reported in [3]. From the analysis a 70 million population from 38 countries, the global prevalence of NAFLD was estimated to be 30.2% [4]. In India, the prevalence of NAFLD is estimated at 38%, with rates reaching more than 60% in urban areas [5], with 1.59% higher risk of developing the disease in males than females, as per the study reported in [6].

The disease burden also extends beyond health outcomes, with patients and families experiencing financial stress due to chronic liver disease management [7]. The analysis carried out for the period from 1990 to 2021 shows that NAFLD-related cirrhosis is rising, with about 1.43 million deaths due to cirrhosis and chronic liver diseases in 2021 [8]. These findings emphasize the need for automated tools for liver disease detection and classification.

Liver biopsy is the gold standard for fibrosis assessment; however,  it is invasive, costly, and prone to sampling errors, patient discomfort, and inter-observer variability [9,10]. This has motivated the development of non-invasive methods such as elastography (FibroScan) and serological biomarkers [11,12]. However, these methods have the issue of limited reproducibility and performance across diverse populations. Ultrasound imaging is safe, low-cost, and widely accessible, making it an ideal modality for developing automated diagnostic systems for liver evaluation. However, its diagnostic sensitivity for early-stage fibrosis remains modest and is affected by speckle noise, low contrast, and inter-device variability.

Recently, deep learning (DL) has been widely used for liver disease classification [13,14,15]. Convolutional neural networks (CNNs) have shown strong potential in learning discriminative features directly from raw medical images. Kagadis et al. [16] used a DL network with fine-tuned elastrography image sequences for liver disease assessment. A DL-based framework for liver cancer histopathology image classification is presented in [17] using CNNs trained with global labels only. A multi-scale CNN for liver disease classification in ultrasound images proposed by [18] achieved an accuracy above 90% and an AUROC of 97.8%. The effect of dataset size on histopathology-based hepatocellular carcinoma (HCC) classification is investigated by [19]. The DL classifier achieved over 90% accuracy, sensitivity, and specificity in HCC classification.

Subsequent studies have increasingly integrated advanced AI methodologies with ultrasound imaging. Ultrasound radiomics was used to detect early-stage fibrosis, demonstrating the utility of handcrafted imaging biomarkers with improved accuracy [20]. A generative adversarial network (GAN) was investigated by [21] for ultrasound-based fibrosis classification, combining synthetic data generation with a radiomics nomogram. An ensemble of deep learning networks was used on heterogeneous ultrasound images acquired across imaging devices to classify liver fibrosis [22]. A modified Faster R-CNN framework is proposed by [23] for ultrasound-based diagnosis of liver diseases, which achieved strong diagnostic performance but required high computational resources.

Ai et al. [24] used frequency–domain features by applying a one-dimensional CNN model to ultrasound radiofrequency (RF) spectra. Park et al. [25] evaluated CNNs for the automated classification of fibrosis in B-mode ultrasound, reporting accuracies above 90% and reasonable AUROC. These advancements demonstrate the increasing maturity of CNN-based solutions for fibrosis classification. More recently, hybrid frameworks have been widely used. Le et al. [26] explored radiomics as a paradigm shift in liver disease detection and classification. Xia et al. [27] proposed a method for diagnosing fibrotic non-alcoholic steatohepatitis (NASH) using an ultrasound radiomics-based logistic regression model.

Despite significant progress, several challenges remain. Histopathology-based approaches achieve strong accuracy but are invasive and impractical for repeated monitoring. CNN-based ultrasound models show excellent performance but are sometimes limited by generalizability across institutions and imaging devices. Radiomics approaches enhance interpretability but often rely on handcrafted features, which may limit scalability. GAN-based augmentation helps mitigate data scarcity but raises questions about clinical reliability.

In this study, we are interested in a situation where labelled data is available in relatively small quantities, and we are computation-constrained as well. To combat this, we propose a framework, *LivSCP*, that is useful in such data- and compute-constrained cases. Also, our method aims to boost the slow-improving performance of the liver fibrosis classification models with time.

Supervised learning (SL), unsupervised learning (UL), reinforcement learning (RL), and self-supervied learning (SSL) are common paradigms to learn model(s) from the available data. The most common, regular SL in data-constrained conditions might cause overfitting or data ill-separability. For the situations where, although in lower quantities, labelled data is available, using UL will not make the best use of the available labels. RL has many limitations; the most significant one is computation. RL requires multiple models and good computing power to post-train a model; however, it is not feasible to use it in the training phase. SSL is an interesting paradigm with which we can pre-train models on a corpus of data without labels. For vision models, methods like DINO, MAE, etc., are common choices. However, SSL is also not suitable for our case, where we are limited both by data and computational capability. Also, learning by reconstruction, like in MAE, does not lead to very well-transferable weights [28].

## 2. Materials and Methods

**Figure 1:**
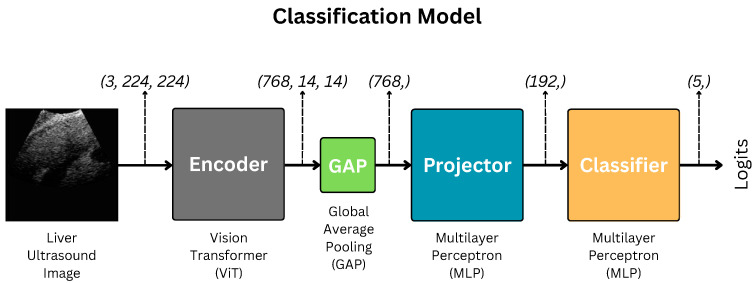
Architecture of the liver fibrosis classification model. GAP indicates Global Average Pooling.

We use the Vision Transformer-based classification model illustrated in Figure 1 for this study. The methods, data, and architectures are detailed well in this section.

### 2.1. Data

**Figure 2:**
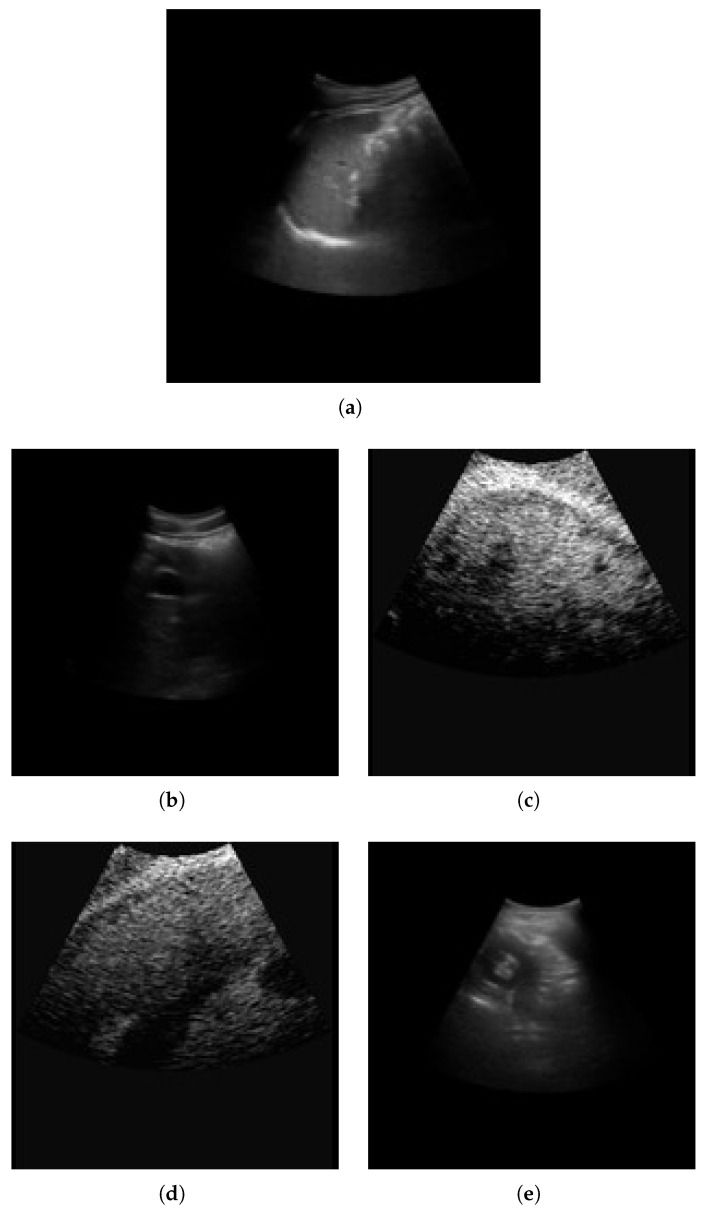
Sample liver images across fibrosis stages (F0–F4). (**a**) F0; (**b**) F1; (**c**) F2; (**d**) F3; (**e**) F4.

The METAVIR score is used for fibrosis classification. The stages are classified as F0, F1, F2, F3, and F4. F0 is the normal tissue with no fibrosis and no architectural distortion. F1 is portal fibrosis without septa. It is symptomatic and may be reversible. F2 is termed periportal fibrosis. Here, fibrosis extends beyond portal tracts, forming thin septa. This indicates significant fibrosis associated with a mild increase in liver stiffness. F3 is termed septal fibrosis with fibrotic septa linking portal tracts and central veins. In this stage, there is substantial distortion of hepatic architecture; however, cirrhosis not fully developed. F4 is the last stage of cirrhosis, with extensive fibrosis with regenerative nodules and complete architectural distortion. It is associated with complications like portal hypertension, liver failure, and hepatocellular carcinoma. The sample liver images across fibrosis stages (F0–F4) are shown in Figure 2.

The liver images for fibrosis classification are sourced from Kaggle [29]. The dataset contains a total of 6323 images. The distribution of images among the stages is detailed in Table 1.

The images are resized to a uniform size of (224,224) through bicubic interpolation [30] and normalized via Z standardization. After this, we split the data into three sets—training, validation, and testing—at a ratio of 8:1:1. Data in the same quantities and proportions were used during training and testing. We use no additional class-balancing methods since our method handles this inherently.

Unlike other methods, we use much simpler augmentations, specifically 90° rotation, horizontal and vertical flipping, color jitter, blurring, and noising. Classic SupCon [31] uses RandAugment [32] to create augmentations of the training images.

### 2.2. Encoder: Vision Transformer

We use Vision Transformer (ViT) [33] as the feature backbone to build the classification models. ViT is based on the transformer architecture [34], which was originally proposed for language applications. Transformers use multihead attention to learn long-range dependencies in the data. They are excellent at learning a robust global representation of the data and better at generalizability than Convolutional Neural Networks (CNNs). In ViT, the input image is first divided into non-overlapping patches; this process is called patch embedding. Considering input $X\in R^{H\times W\times C}$, the patched image $X_{p}\in R^{N\times (P^{2}\cdot C)}$ is obtained as follows:

(1) $$X_{p}=\mathrm{PatchEmbedding}\left(X\right)$$

where PatchEmbedding is a convolutional layer (in practice) and *N* is the number of patches given by the following:

(2) $$N=\frac{H\cdot W}{P^{2}}$$

where *P* is the size of a square patch.

The model card from [35] is used to carry out all our experiments. We use the ViT encoder only, discarding the rest, and build smaller projection and classifier heads. The pretrained encoder is completely frozen after SCP, which is different in the case of SupCon [31], as it keeps the encoder trainable throughout.

We prefer ViT over CNN-based models for the encoder due to its superior continual learning performance [36,37,38]. This enables better knowledge retention from SCP. As we aim to improve the fibrosis classification performance through SCP, it is crucial to choose an encoder that demonstrates better continual learning performance, which the ViT does. Hence, it is preferred for this study.

### 2.3. Projection and Classification Heads

The encoding process can be formulated as follows:

(3) f=Encoder(x)

where $f\in R^{768\times 14\times 14}$ signifies the encoded feature map, Encoder signifies the ViT encoder, and $x\in R^{3\times 224\times 224}$ is the input image.

Then, *f* after Global Average Pooling, GAP, is fed to the projection head, Projector, to obtain projected features *m*. Mathematically,

(4) m=Projector(GAP(f))

as per our implementation, $\mathrm{GAP}\left(f\right)\in R^{768}$, and $m\in R^{192}$.

Finally, *m* is fed to the classifier to obtain the logit *z*.

(5) z=Classifier(m)

where $z\in R^{C}$. *C* is the number of classes; thus, $z\in R^{5}$ in our case.

**Figure 3:**
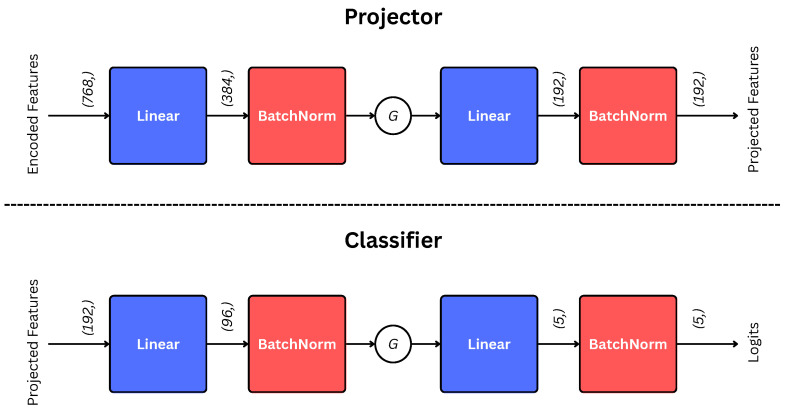
Architectures of projection and classification Heads. The *G* indicates GELU activation [39]. The encoded features (input to the projector) are first pooled through GAP, as illustrated in Equation (4).

Projector and Classifier are shallow multilayer perceptrons (MLPs). Their architectures are illustrated in Figure 3.

We freeze the encoders while training the projection and classification heads.

### 2.4. Supervised Contrastive Learning

SCL [31] aims to maximize the inter-class distance and minimize the intra-class distance in the embedding space. This is achieved by using a Supervised Contrastive Loss function for a single sample *i* given by the following:

(6) $$L_{i}^{\sup}=\frac{-1}{\left|P(i)\right|}\sum_{p\in P(i)}\log\frac{\exp(z_{i}\cdot z_{p}/\tau )}{\sum_{a\in A(i)}\exp\left(z_{i}\cdot z_{a}/\tau \right)}$$

where P(i) is the set of indices of all possible positive samples, A(i) is the set of all possible contrastive samples, τ is temperature, $z_{i}$ is the feature embedding vector of the anchor sample, $z_{p}$ is the feature embedding vector of a positive sample, and $z_{a}$ is the feature embedding vector of any sample a∈A(i). And, it is averaged over all samples in the batch as follows:

(7) $$L^{\sup}=\frac{1}{N}\sum_{i=1}^{N}L_{i}^{\sup}$$

The encoder is pretrained with SCL in LivSCP. To combat the problem of class imbalance, as mentioned in Table 1, we use Class-Weighted Supervised Contrastive Loss (CW-SCL). CW-SCL extends the standard SCL by incorporating class-specific weights $w_{y_{i}}$ to handle class imbalance. This is defined as follows:

(8) $$L^{\mathrm{cw}\text{-}\sup}=\frac{1}{N}\sum_{i=1}^{N}\frac{w_{y_{i}}}{\bar{w}}L_{i}^{\sup}$$

where $\bar{w}=\frac{1}{N}\sum_{i=1}^{N}w_{y_{i}}$ is the mean class weight, and $L_{i}^{\sup}$ is the supervised contrastive loss for sample *i* given by the following:

(9) $$L_{i}^{\sup}=\frac{-1}{\left|P(i)\right|}\sum_{p\in P(i)}\log\frac{\exp(z_{i}\cdot z_{p}/\tau )}{\sum_{a\in A(i)}\exp\left(z_{i}\cdot z_{a}/\tau \right)}$$

Substituting, the complete expression for the CW-SCL becomes the following:

(10) $$L^{\mathrm{cw}\text{-}\sup}=-\frac{1}{N}\sum_{i=1}^{N}\frac{w_{y_{i}}}{\bar{w}\;\left|P\left(i\right)\right|}\sum_{p\in P(i)}\log\frac{\exp(z_{i}\cdot z_{p}/\tau )}{\sum_{a\in A(i)}\exp\left(z_{i}\cdot z_{a}/\tau \right)}$$

The class weights are computed inversely proportional to the class frequencies to handle imbalance, defined as follows:

(11) $$w_{c}=\frac{1/n_{c}}{1/C\sum_{k=1}^{C}1/n_{k}}$$

where $n_{c}$ is the number of samples in class *c*, and *C* is the total number of classes. This normalization ensures that the mean of all class weights is 1.

*LivSCP* includes pretraining the encoder with CW-SCL and then training the projection and classification heads on the embeddings produced by the frozen pretrained encoder with SL.

## 3. Results

### 3.1. Quantitative Evaluation

**Figure 4:**
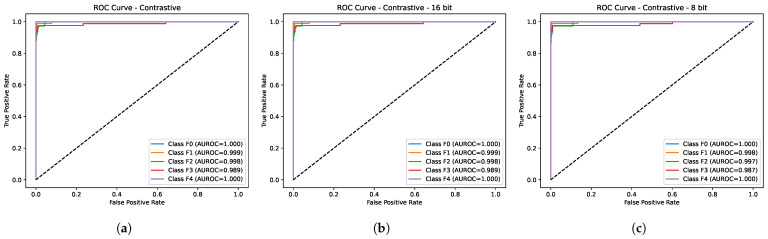
ROC curves for models trained through LivSCP. (**a**) FP32. (**b**) FP16. (**c**) INT8.

**Figure 5:**
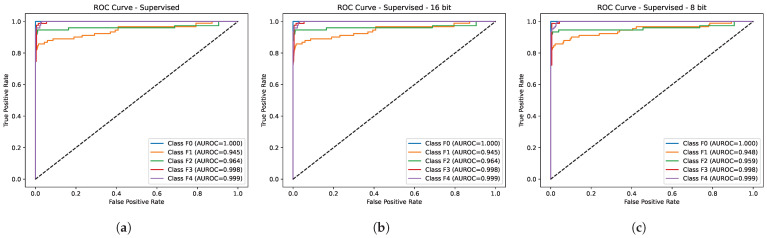
ROC curves for models trained through SL. (**a**) FP32. (**b**) FP16. (**c**) INT8.

Table 2 depicts the comparative performance of the proposed method (LivSCP) with SL at different quantization levels across various metrics. As observed from Table 2, there is an absolute improvement of 5.38% in accuracy and recall, 3.88% in precision, 5.62% in F1-score, and 1.6% in mAUROC with the proposed method LivSCP as compared to SL in FP32 precision. The ROC Curves for models trained through LivSCP and SL for FP32, FP16, and INT8 precision are shown in Figure 4 and Figure 5, respectively.

From here, figures in red and green indicate the lowest and highest values of the evaluation metrics, respectively.

### 3.2. Implementation Details

We use PyTorch (version 2.6.0) to implement, train, and test the models. NVIDIA P100 GPUs are used for accelerated training and inference. Since older GPUs do not support INT8 inference, CPUs are used for inference in Table 2. All convolutional layers are initialized using Kaiming Initialization [40], while the linear layers are initialized using Xavier Initialization [41]. The scale and shift parameters in the BatchNorm layers are initialized with ones and zeros, respectively. AdamW optimizer [42] is used while training all the models. The classes MulticlassAccuracy, MulticlassPrecision, MulticlassRecall, MulticlassF1Score, MulticlassAUROC, and MulticlassROC from torchmetrics are used to compute the performance metrics. Our source code, trained models, and results are publicly available at https://github.com/adityabhongade/LivSCP/ (accessed on 6 December 2025).

For pretraining the encoder in LivSCP, we use temperature τ=0.07, learning rate $\eta =3\times 10^{-4}$, and L2 weight decay $\lambda =10^{-4}$. The encoder is (pre)trained for a maximum of 50 epochs with a custom implementation for early stopping. These values were obtained via a small grid-search method due to computational constraints. GridSearchCV class was used, as provided in the model_selection module from the sklearn library.

## 4. Discussion

### 4.1. Contribution

This work primarily aims to improve the slow-growing performance of liver fibrosis classification methods. We make the following contributions to the application with this work:

- **Improved performance:** As seen above, our method, *LivSCP*, improves classification performance without any changes to the network architecture of ViT. It outperforms all existing methods and our baseline (ViT with SL) consistently across all evaluation metrics.
- **Solution to Low Data and Computation:** Our method is successful in low-data and -computation settings. We have demonstrated that with a dataset of 6323 images in total (∼5058 images for training), and an NVIDIA P100 GPU, we found an absolute performance boost of up to 5.38% in accuracy with respect to our baseline and up to 14.93% with an existing method [22].
- **Rigorous performance analysis:** We conduct a rigorous quantitative analysis of the classification performance of existing methods, our baseline, and the proposed method at different precision (quantization) levels.

### 4.2. Comparison with Baseline

We consider ViT trained with SL as our baseline. We simultaneously implement this baseline along with our method with the same training settings.

In Table 2, a comparison of the classification performance of the proposed method and baseline is illustrated at different precision levels. It can be seen that INT8 performance is generally better than that of the FP32 and FP16, especially in the case of SL. This is a classic case of quantization acting as regularization [43]. We can observe that LivSCP consistently outperforms SL across all precision levels and evaluation metrics. There is an improvement of an absolute 5.38% in accuracy. This is a remarkable improvement since the network architectures, learning rate, initial weights, and optimizer are all the same.

### 4.3. Comparison with Existing Methods

A comparison of diagnostic performance for liver fibrosis classification using the proposed method with existing methods on the basis of accuracy and AUROC is given in Table 3. Lee et al. [13] reported an accuracy of 83.5 and 76.4 % on internal and external datasets of four classes of liver fibrosis with an AUROC of 0.901 and 0.857 for F4 stage classification. Joo et al. [22] reported a mean accuracy of 84.37% and Park et al. [25] reported a mean accuracy and mean AUROC of 0.95 for liver disease classification.

The comparison of classification accuracy obtained using the proposed network with five DL networks from [22] is given in Table 4. ResNet and EfficientNet showed strong performance with an accuracy of 85.92% and 85.17%, respectively. However, the proposed method reported an accuracy of 98.10%, indicating significant improvement in classification performance and demonstrating the effectiveness of the proposed method over existing DL methods.

Table 5 shows the AUROC values obtained for liver fibrosis classification across stages (F0–F4) using our proposed method and five DL networks from [25]. The proposed method demonstrates superior performance across all fibrosis stages with AUROC values of 1.000 for F0 and F4, 0.999 for F1, 0.998 for F2, and 0.989 for F3, indicating that it achieved better discrimination across all fibrosis stages.

### 4.4. Implications

There are several technical and non-technical (social and human) implications of using LivSCP in clinical applications.

- **Explainability:** DL models used for automated disease classification are no less than unexplained black boxes. LivSCP, being one, will have similar characteristics, and we might question its interpretability. This creates immense scope for further research in developing solutions that are inherently explainable and do not need external methods to explain them, such as SHAP [44], GradCAM [45], etc.
- **Case-specificity:** LivSCP has been developed to address scenarios where both data and computational resources are limited. However, its performance may vary and may even improve when more data and greater computing capacity are available. Therefore, our method should not be generalized to broader studies without considering these constraints.
- **Practical Errors:** LivSCP has no solution to reduce the effect of imaging errors and noise, inconsistencies, etc. This creates another opportunity to develop methods that are more error-immune.

## 5. Conclusions and Future Scope

### 5.1. Conclusions

Through our experiments, we found that our method, *LivSCP*, delivers superior performance compared to existing methods and our baseline. The performance gains are consistent across all metrics and precision levels. The proposed method achieves a state-of-the-art performance, with an accuracy, precision, recall, and F1-score of 98.10% each, and an AUROC of 0.9972. A major advantage of LivSCP is that it does not require any modification to the network architecture. Our method is particularly well-suited for scenarios with limited labeled data and computational resources. Therefore, we propose a sophisticated approach for training liver fibrosis classification models that achieves high performance even with limited data and computational resources. The proposed method has been compared against various existing techniques and demonstrates strong potential for clinical applications.

### 5.2. Future Scope

The objectives discussed here may be explored in the future to improve the performance, reliability, and adaptability of liver fibrosis classification methods. We believe that with the availability of multiple data modalities for liver fibrosis classification, developing multimodal methods will become significantly easier. This would help improve classification performance as well as adaptability.

In the future, efforts should also focus on developing methods that can handle imaging errors and inconsistencies more effectively, since these factors strongly influence the efficacy of such systems. Additionally, methods that are inherently explainable should be prioritized. In medical applications, interpretability plays a crucial role in making any system trustworthy. Such inherently explainable systems will also help us gain deeper insights into the diseases themselves.

## Figures and Tables

**Table 1:** Liver fibrosis stages and corresponding number of images.

Stage	# Images
F0	2114
F1	861
F2	793
F3	857
F4	1698

**Table 2:** Comparative performance of the proposed method (LivSCP) with the baseline (SL) at different quantization levels for the ViT-based network illustrated in Figure 1.

Method	Accuracy	Precision	Recall	F1 Score	mAUROC
SL-FP32	0.9272	0.9422	0.9272	0.9248	0.9812
LivSCP-FP32 ^†^	0.9810	0.9810	0.9810	0.9810	0.9972
SL-FP16	0.9272	0.9422	0.9272	0.9298	0.9811
LivSCP-FP16	0.9810	0.9810	0.9810	0.9810	0.9972
SL-INT8	0.9287	0.9432	0.9287	0.9312	0.9807
LivSCP-INT8	0.9810	0.9810	0.9810	0.9810	0.9964

^†^ Proposed. See Appendix A for the details on quantization.

**Table 3:** Comparison of diagnostic performance of different methods for liver fibrosis classification.

Method	Accuracy (in %)	AUROC
Lee et al. [13]	83.5 & 76.4	0.901 & 0.857 (F4)
Joo et al. [22]	84.37	–
Park et al. [25]	94	0.95
**Proposed**	98.10	0.9972

**Table 4:** Comparison of accuracy values obtained using the proposed method with five DL networks from [22].

Network	Accuracy (in %)
VGGNet	83.17
ResNet	85.92
DenseNet	84.17
EfficientNet	85.17
ViT (SL)	83.42
**Proposed**	98.10

**Table 5:** Comparison of AUROC values obtained using the proposed method with five DL networks from [25].

Network	F0	F1	F2	F3	F4
VGGNet	0.96	0.96	0.98	0.94	0.96
ResNet	0.96	0.96	0.97	0.93	0.94
DenseNet	0.95	0.96	0.95	0.94	0.96
EfficientNet	0.96	0.96	0.97	0.94	0.96
ViT (SL)	0.97	0.94	0.96	0.94	0.96
**Proposed**	1	0.999	0.9983	0.98915	1

## Data Availability

The data is available publicly at https://kaggle.com/datasets/vibhingupta028/liver-histopathology-fibrosis-ultrasound-images (accessed on 6 December 2025). The source code and trained models are public at https://github.com/adityabhongade/LivSCP/ (accessed on 6 December 2025).

## Abbreviations

    The following abbreviations are used in this manuscript:

NAFLD	Non-alcoholic Fatty Liver Disease
ARLD	Alcohol Related Liver Disease
DL	Deep Learning
CNN	Convolutional Neural Network
HCC	Hepatocellular Carcinoma
US	Ultrasound
SWE	Shear Wave Elastography
SL	Supervised Learning
UL	Unsupervised Learning
RL	Reinforcement Learning
SSL	Self-Supervised Learning
SCP	Supervised Contrastive Pretraining
SFT	Supervised Fine-tuning
MLP	Multilayer Perceptron
ViT	Vision Transformer
GAP	Global Average Pooling
mAUROC	Mean Area Under the Receiver Operating Characteristic Curve
CW-SCL	Class Weighted Supervised Contrastive Loss

## Appendix A

### Quantizing Models

We quantize the models to FP16 and INT8 after training or finetuning. For FP16, half() method is used directly. In the case of INT8, quantize_dynamic() method from torch.ao.quantization is used on linear, convolutional, layer normalization, and batch normalization layers [46]. In dynamic quantization, the weights are quantized in advance, while the activations are dynamically quantized during inference.
